# Supplementary figures and images for: Peri-mitochondrial actin filaments inhibit Parkin assembly by disrupting ER-mitochondria contacts
Source: EMBO Rep. 2025 Aug 29;26(20):4977–5008. doi: 10.1038/s44319-025-00561-y (PMC12550048; doi:10.1038/s44319-025-00561-y)

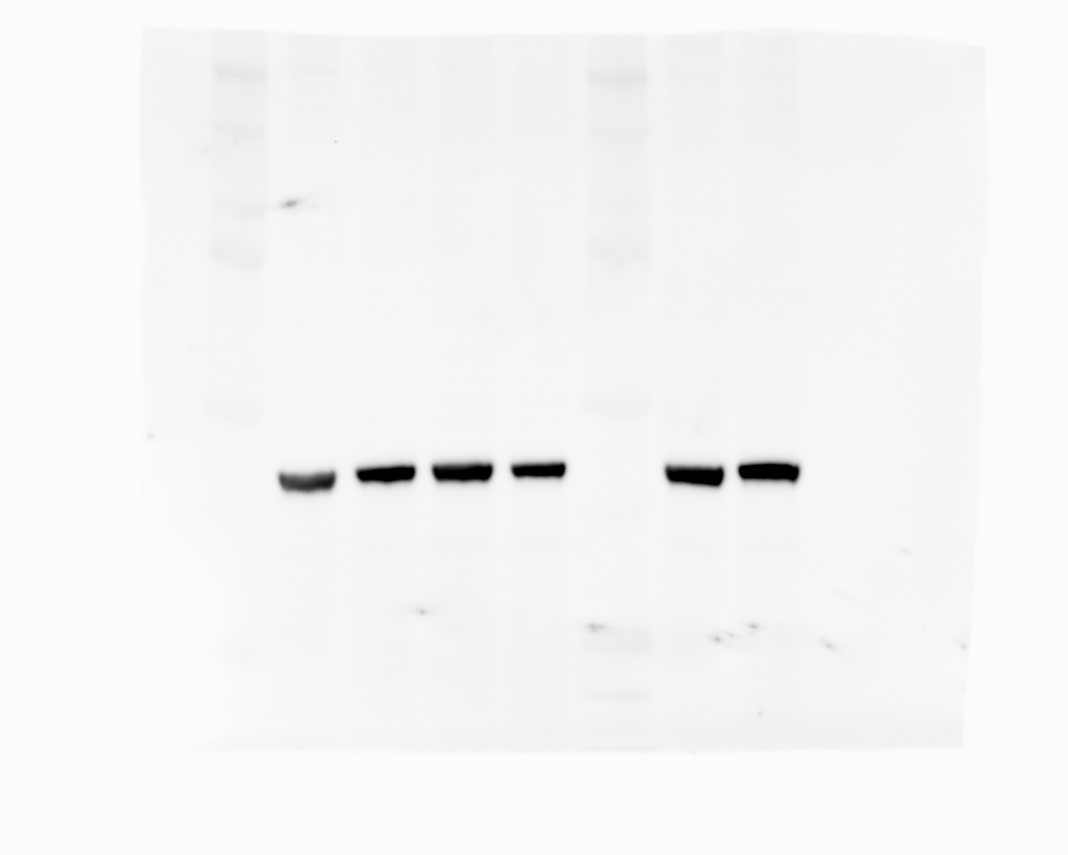

Supplement: Supplementary file 2 — Source data Fig. 4 [file 44319_2025_561_MOESM2_ESM.zip › 4A/CHEMIL_1.TIF]

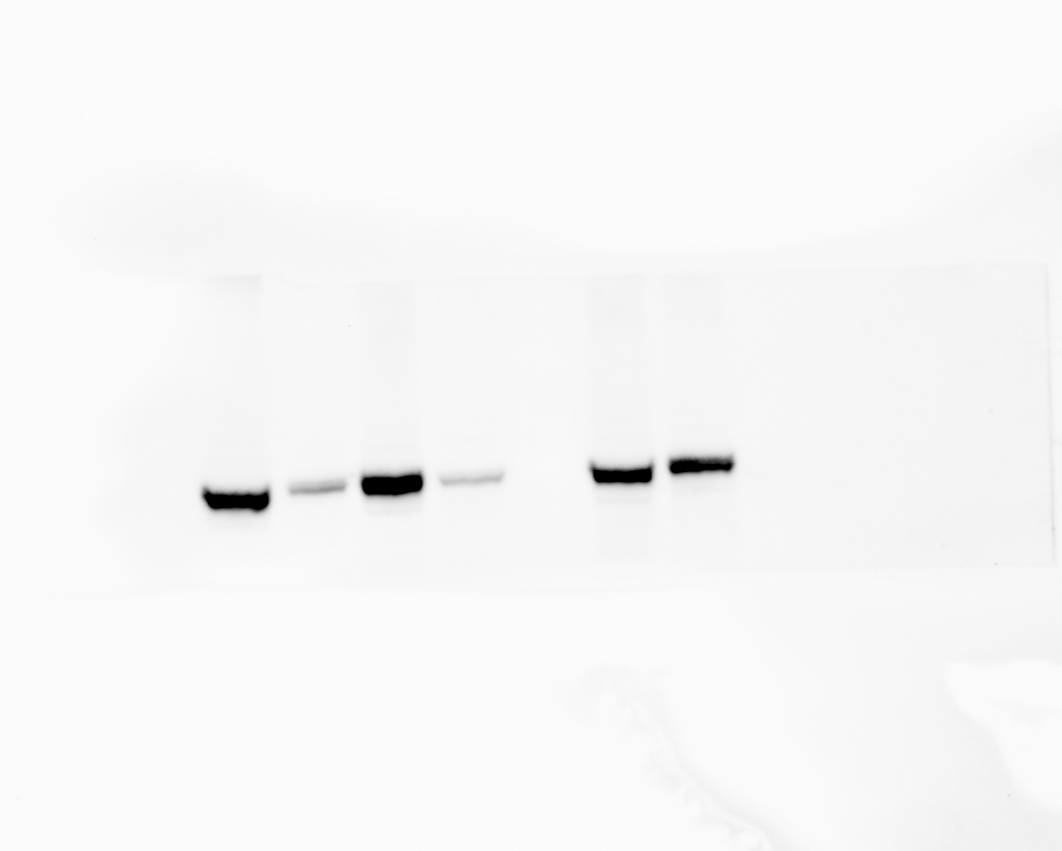

Supplement: Supplementary file 2 — Source data Fig. 4 [file 44319_2025_561_MOESM2_ESM.zip › 4A/Mfn2-Rb-CST_Autoexposed.tif]

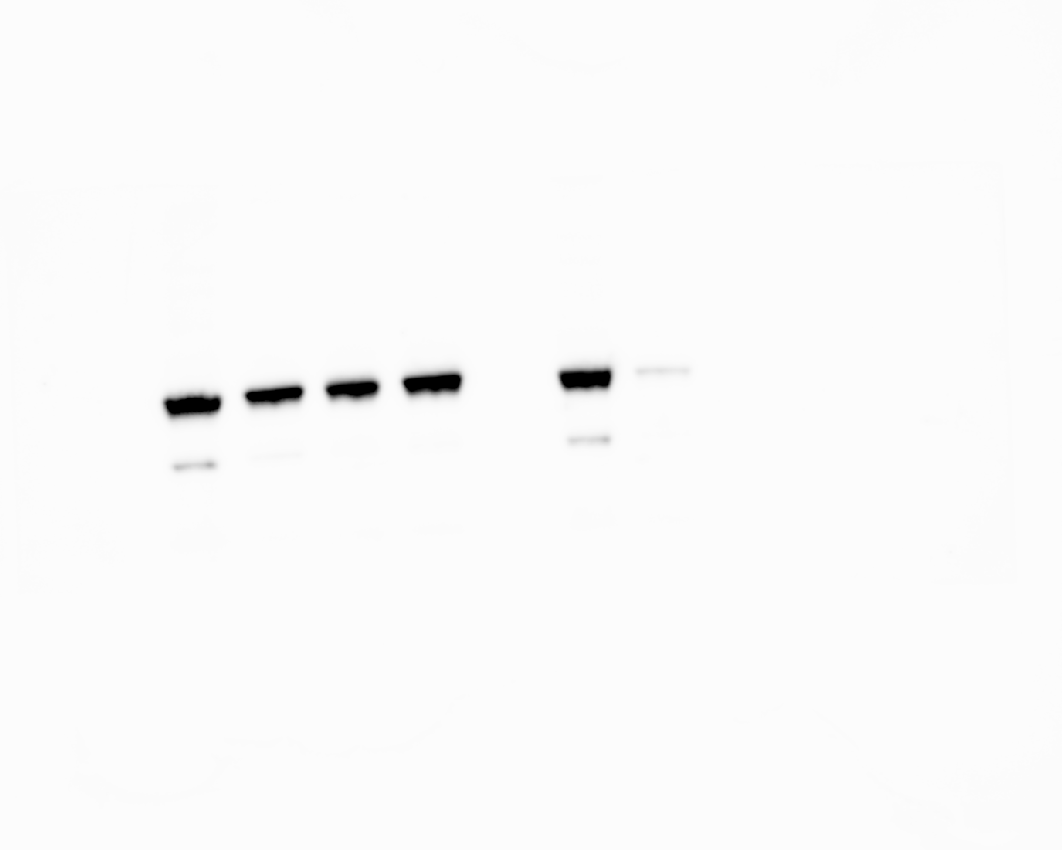

Supplement: Supplementary file 2 — Source data Fig. 4 [file 44319_2025_561_MOESM2_ESM.zip › 4A/VDAC1-Rb-Invitrogen_Autoexposed.tif]

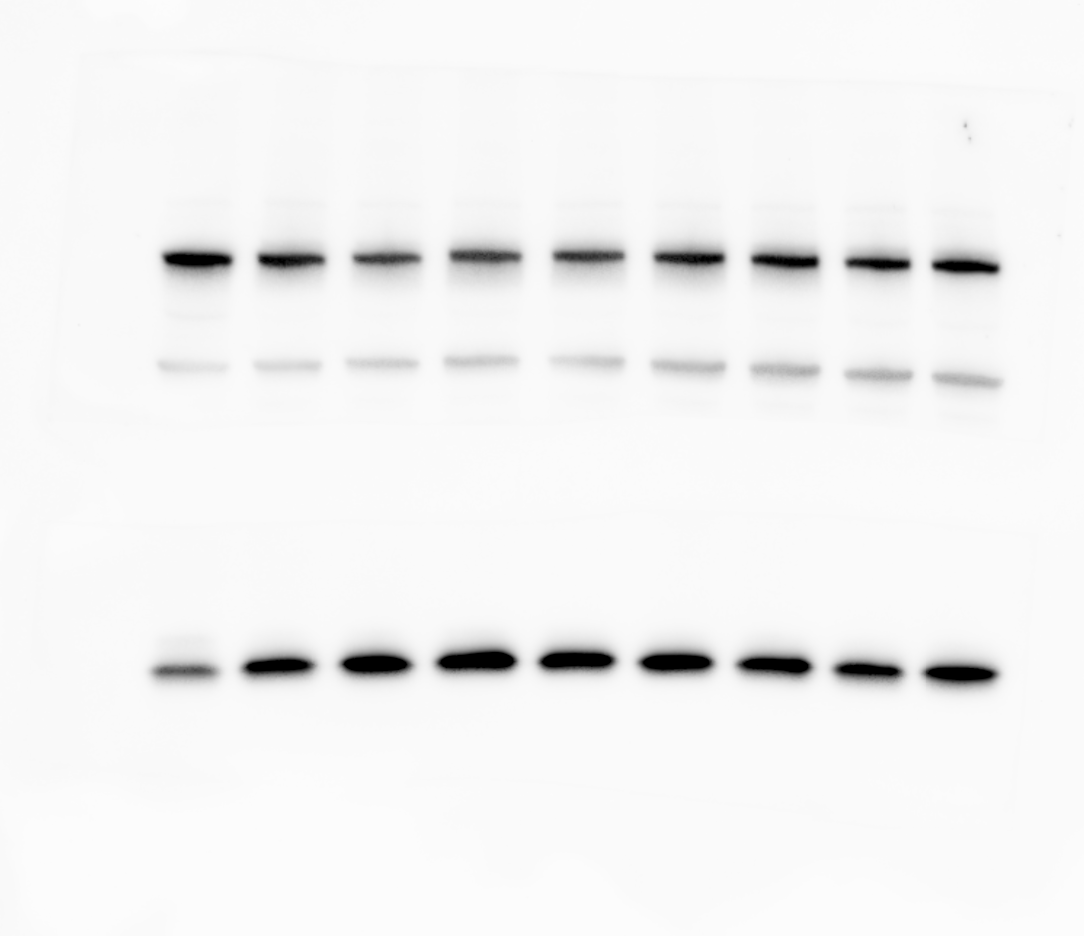

Supplement: Supplementary file 3 — Source data Fig. 5 [file 44319_2025_561_MOESM3_ESM.zip › 5C/Chemiluminescence_2024-11-17_15h49m08s(Chemiluminescence)-1.tif]

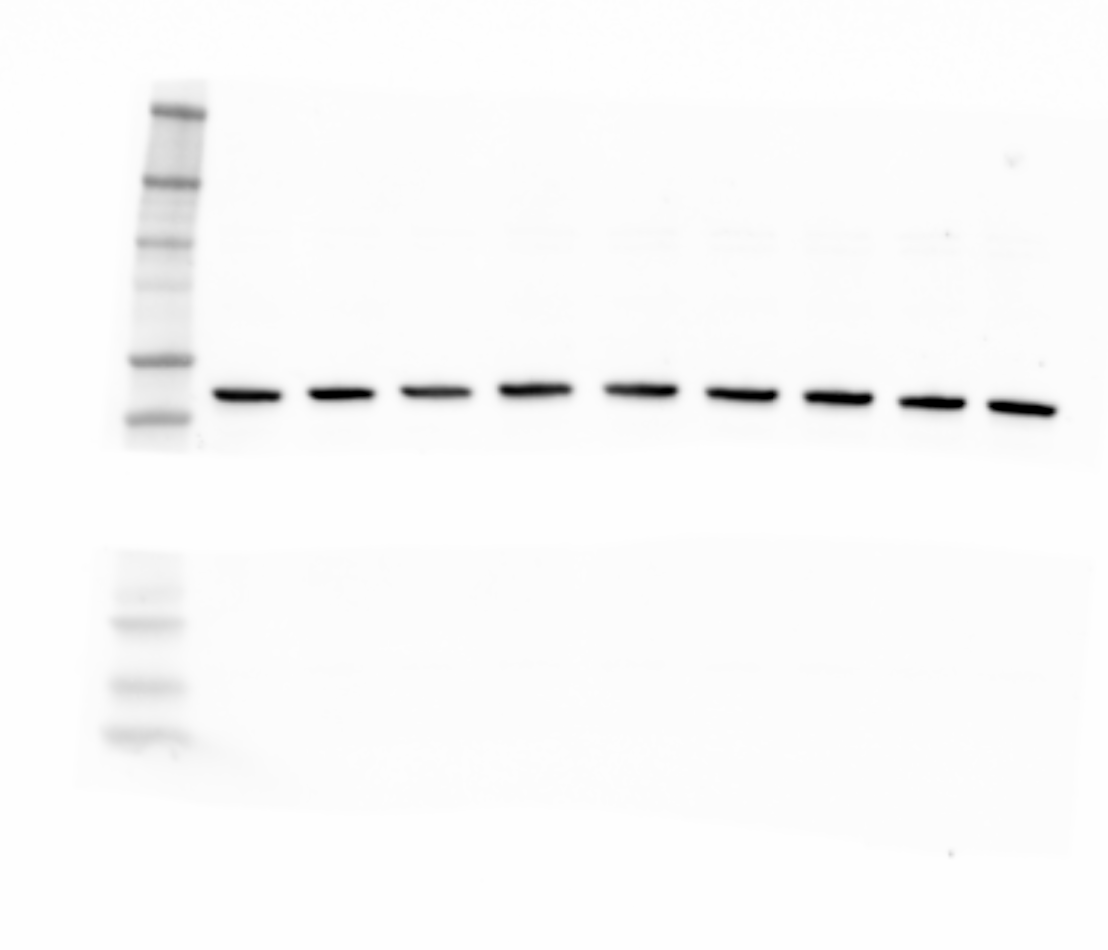

Supplement: Supplementary file 3 — Source data Fig. 5 [file 44319_2025_561_MOESM3_ESM.zip › 5C/IRDye_680RD_2024-11-17_15h39m34s(IRDye_680RD)-1.tif]

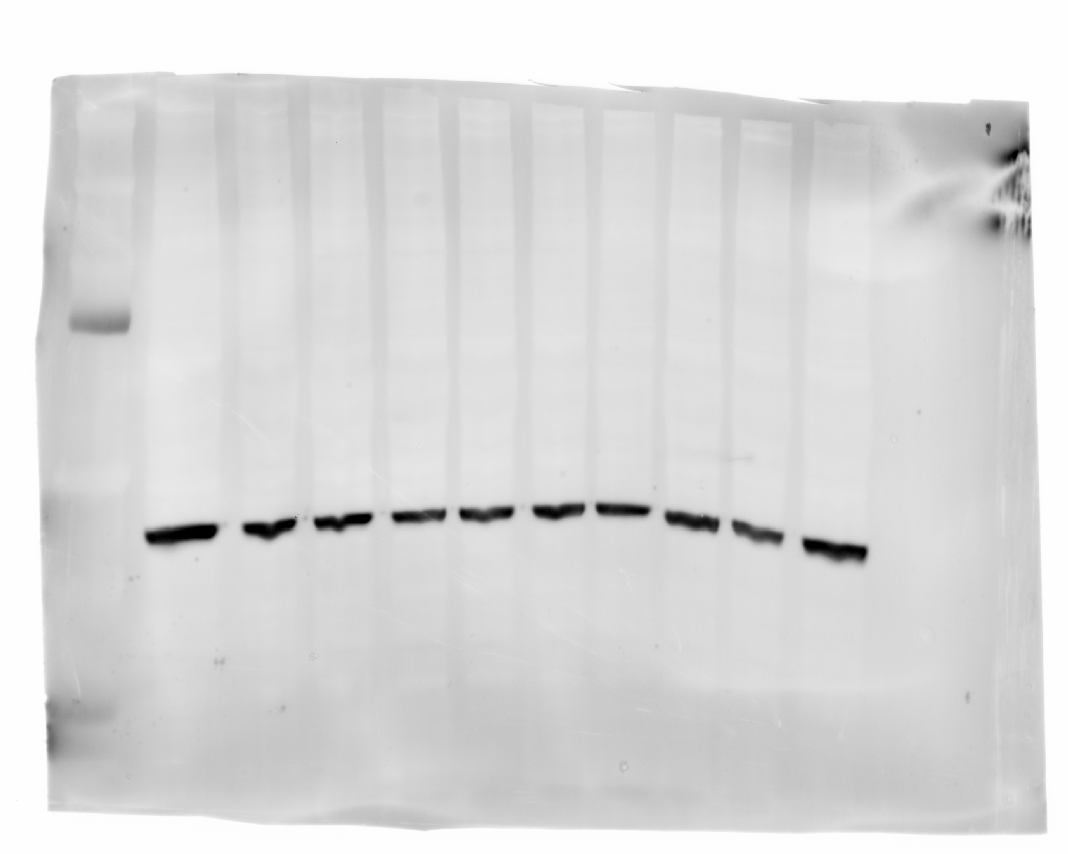

Supplement: Supplementary file 4 — Source data Fig. 6 [file 44319_2025_561_MOESM4_ESM.zip › 6A/6A_actin_StarBright_B700_2025-01-14_12h57m55s(StarBright_B700).tif]

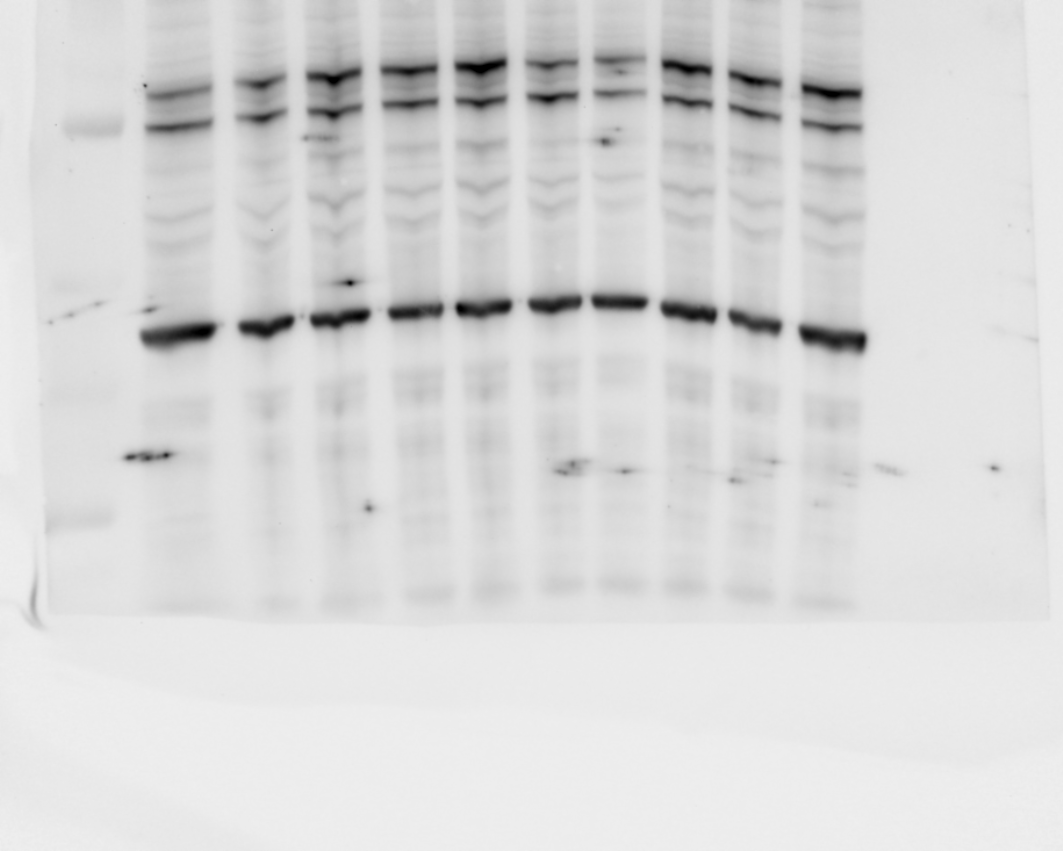

Supplement: Supplementary file 4 — Source data Fig. 6 [file 44319_2025_561_MOESM4_ESM.zip › 6A/6A_Chemiluminescence_2025-01-14_14h02m12s(Chemiluminescence).tif]

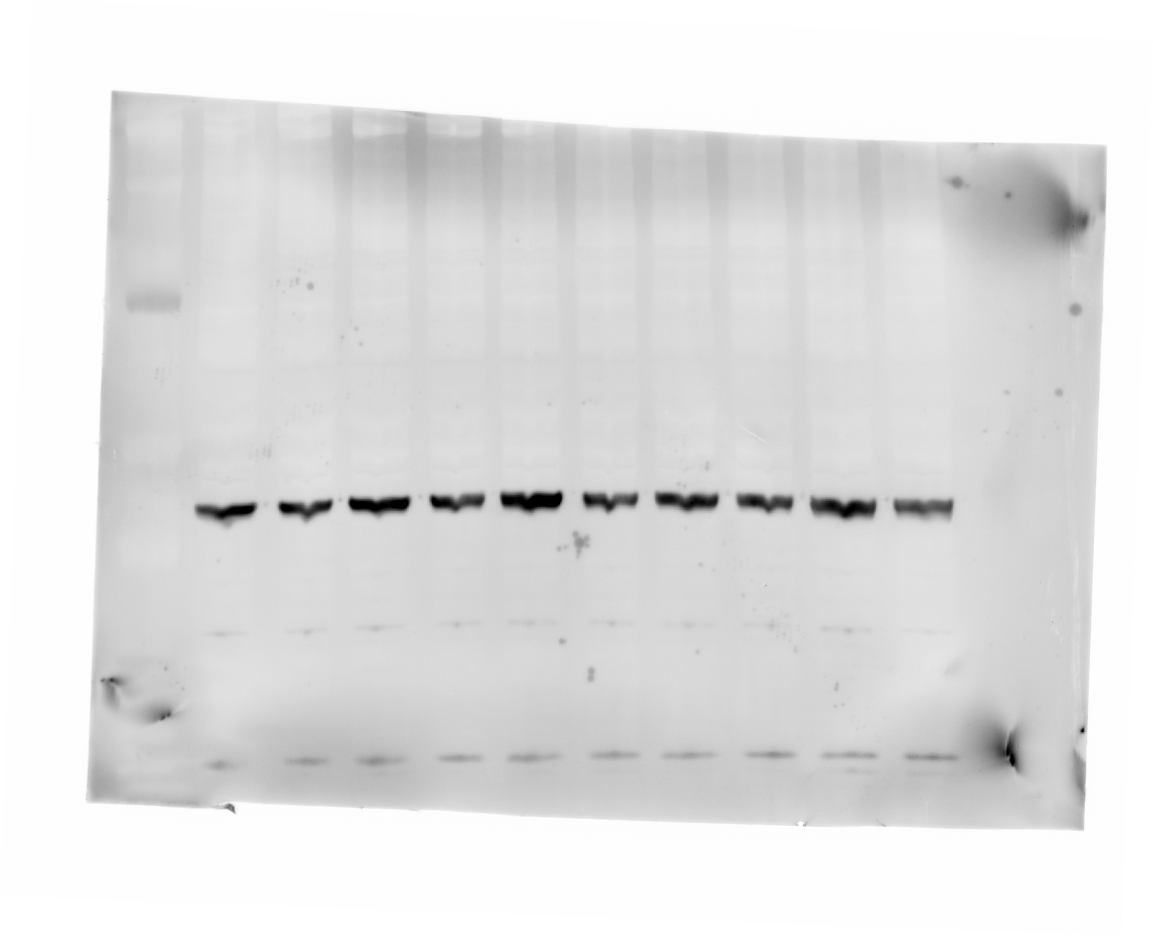

Supplement: Supplementary file 4 — Source data Fig. 6 [file 44319_2025_561_MOESM4_ESM.zip › 6C/6C_actin_Beta_actin-CST-mm.tif]

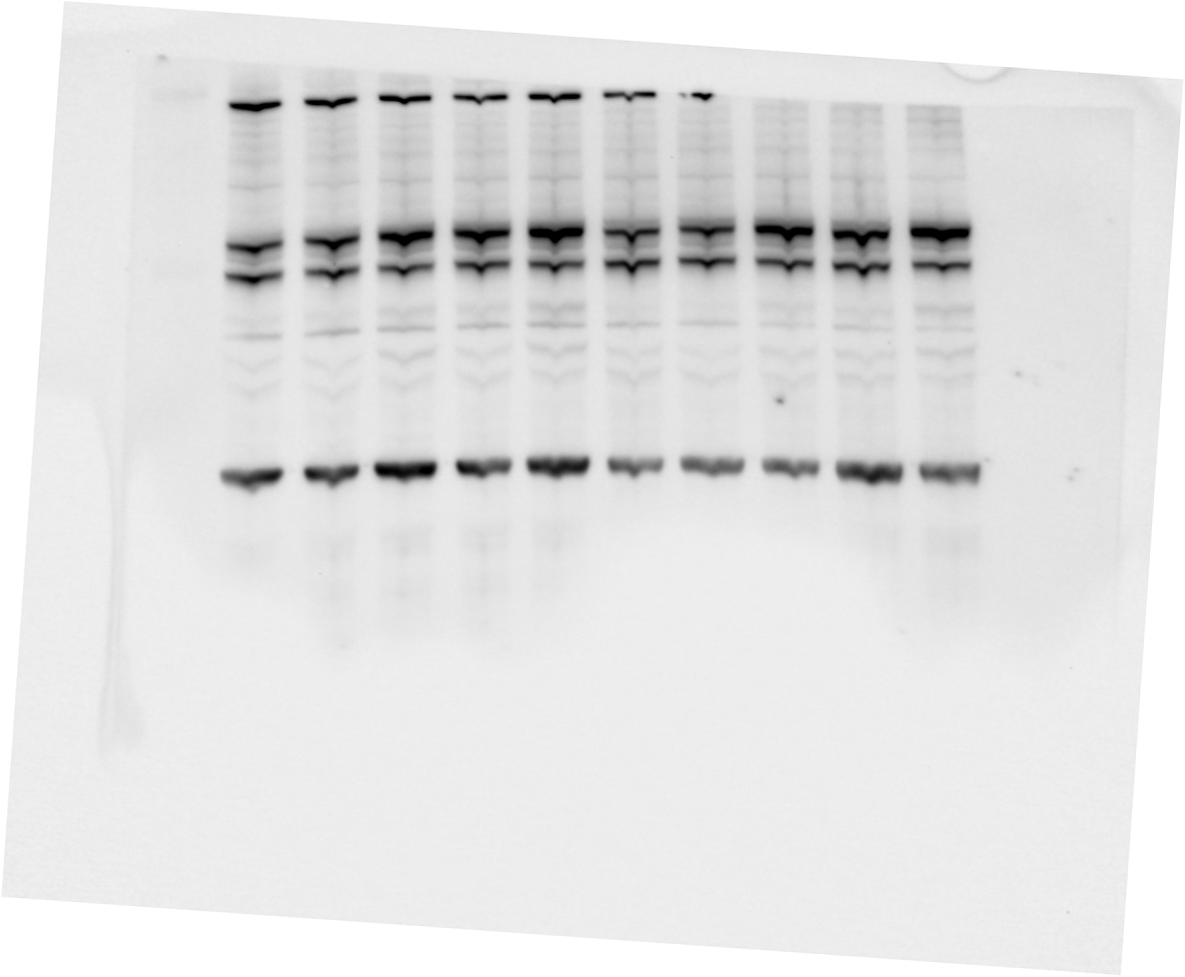

Supplement: Supplementary file 4 — Source data Fig. 6 [file 44319_2025_561_MOESM4_ESM.zip › 6C/6C_Chemiluminescence_2025-01-11_16h40m47s(Chemiluminescence).tif]

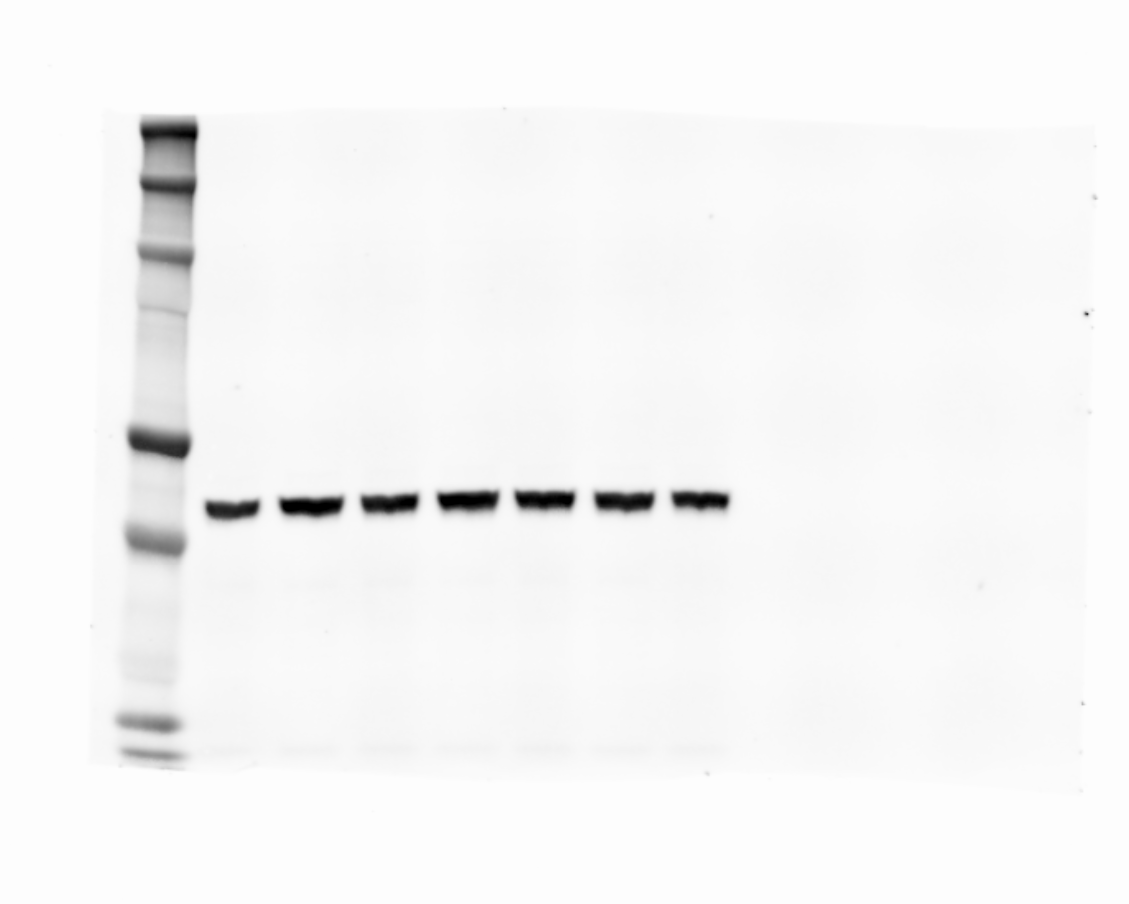

Supplement: Supplementary file 4 — Source data Fig. 6 [file 44319_2025_561_MOESM4_ESM.zip › 6F/Beta_Actin.tif]

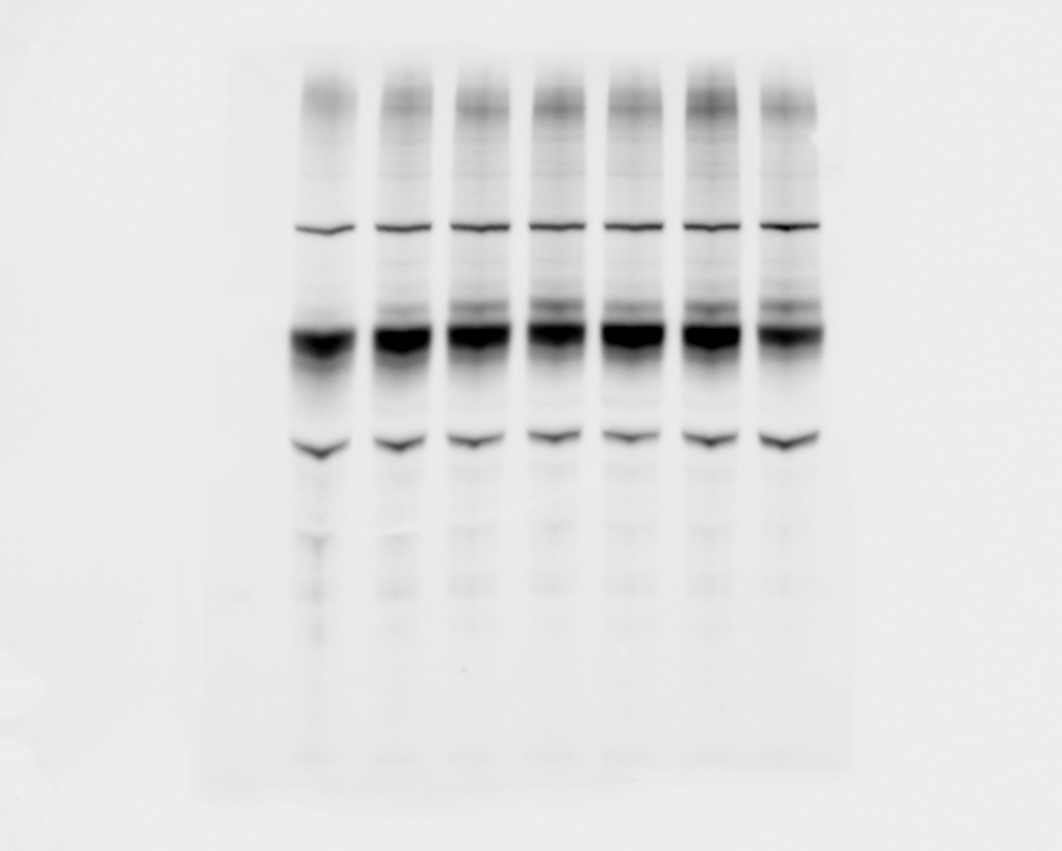

Supplement: Supplementary file 4 — Source data Fig. 6 [file 44319_2025_561_MOESM4_ESM.zip › 6F/CHEMIL_1.TIF]

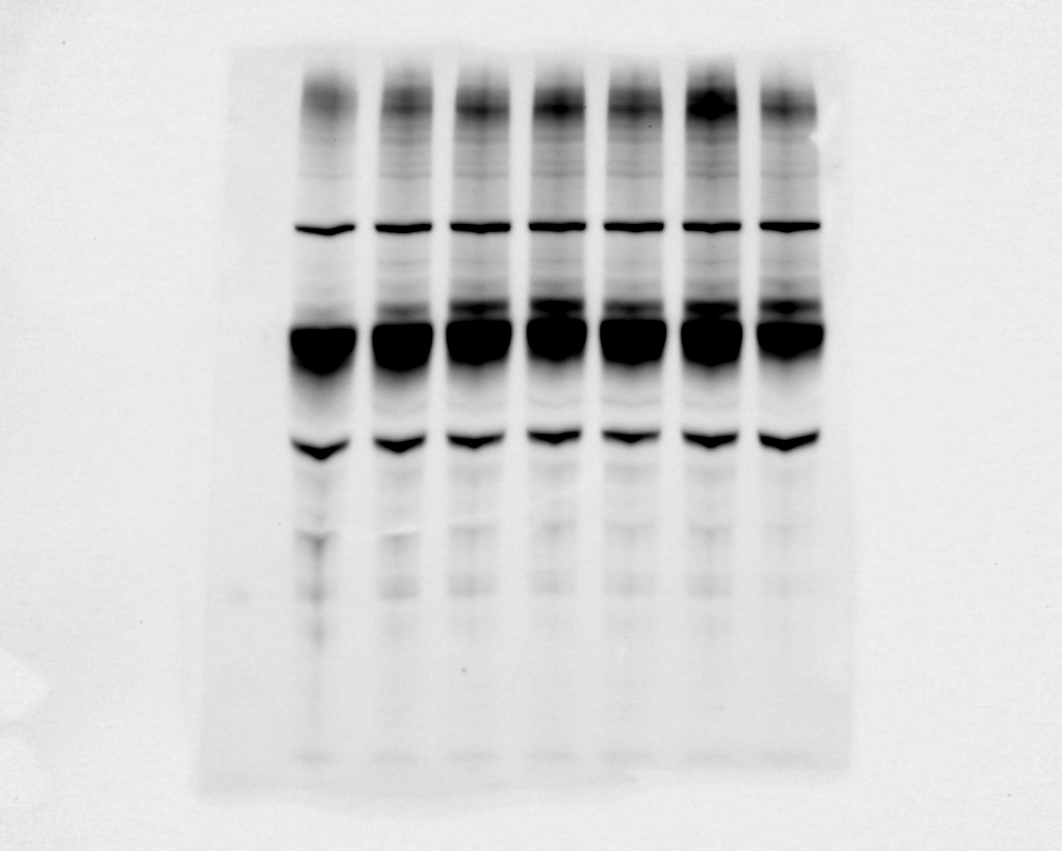

Supplement: Supplementary file 4 — Source data Fig. 6 [file 44319_2025_561_MOESM4_ESM.zip › 6F/CHEMIL_2.TIF]

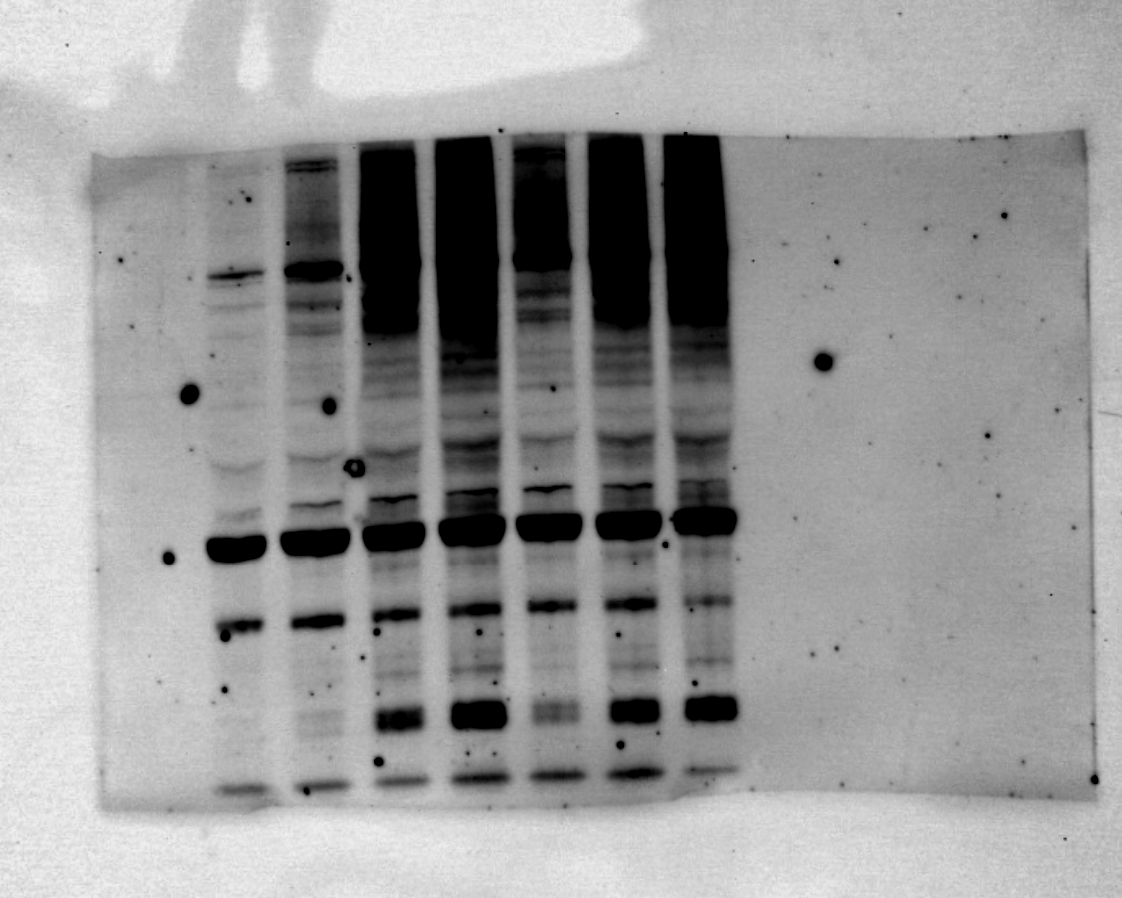

Supplement: Supplementary file 4 — Source data Fig. 6 [file 44319_2025_561_MOESM4_ESM.zip › 6F/CHEMIL_3.TIF]

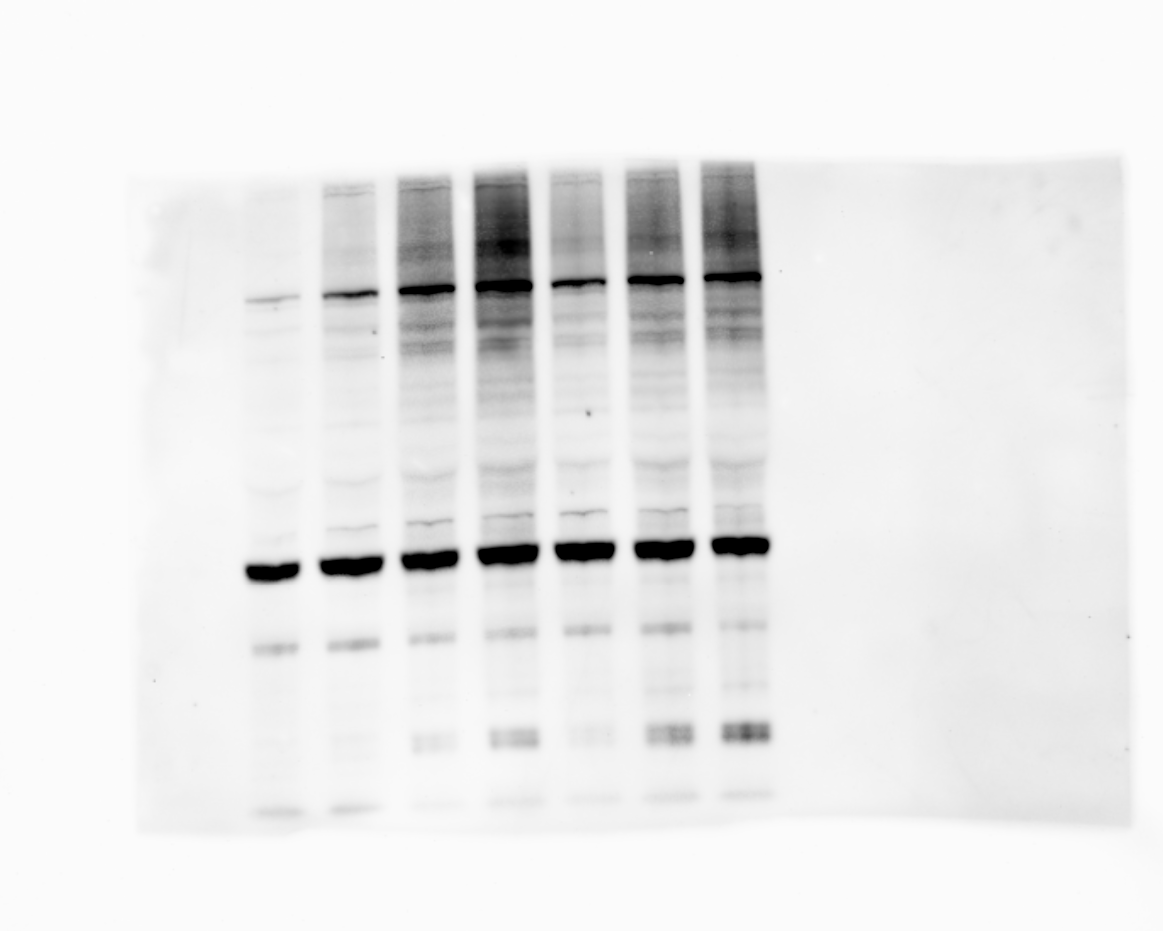

Supplement: Supplementary file 4 — Source data Fig. 6 [file 44319_2025_561_MOESM4_ESM.zip › 6F/Phospho_Ub_Ser65_ORI.tif]

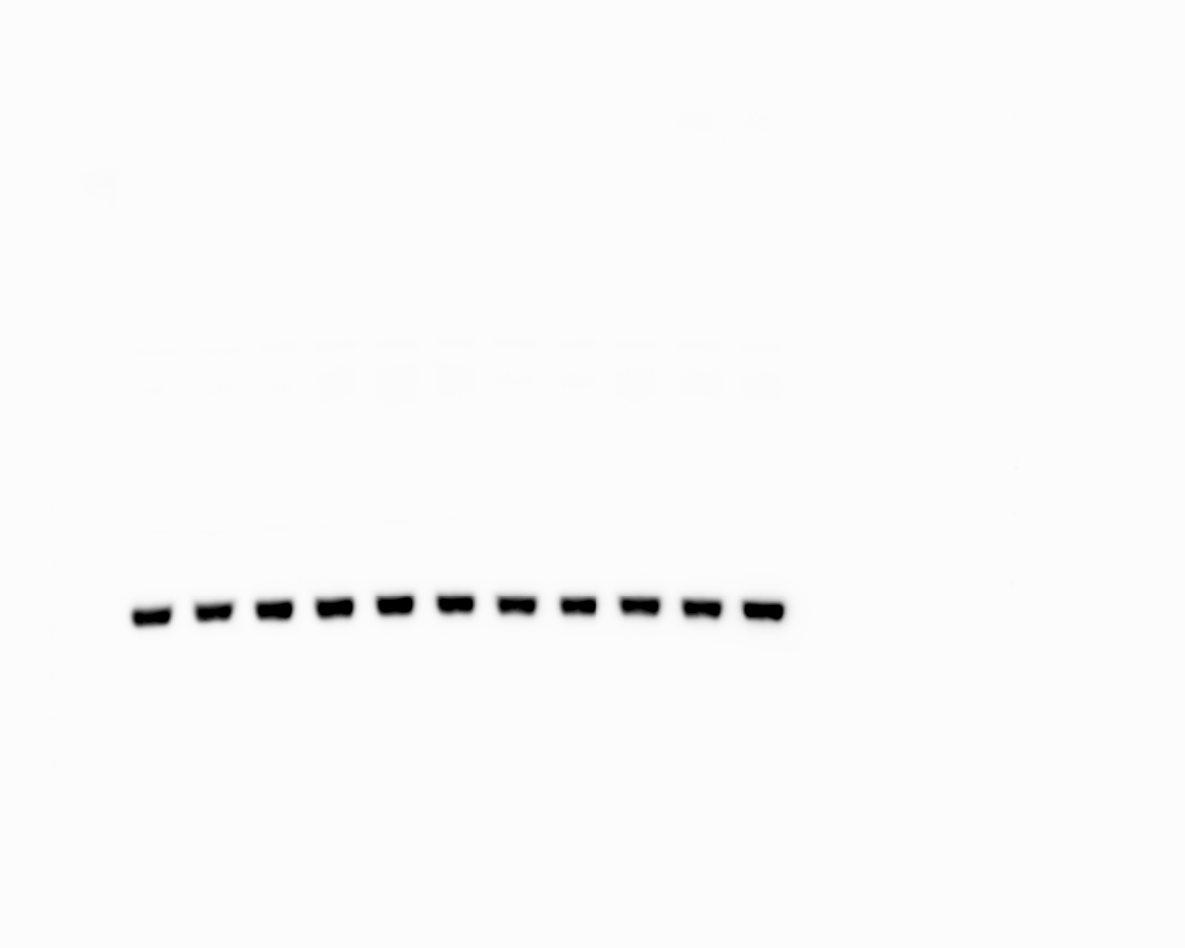

Supplement: Supplementary file 4 — Source data Fig. 6 [file 44319_2025_561_MOESM4_ESM.zip › 6E/Beta_Actin.tif]

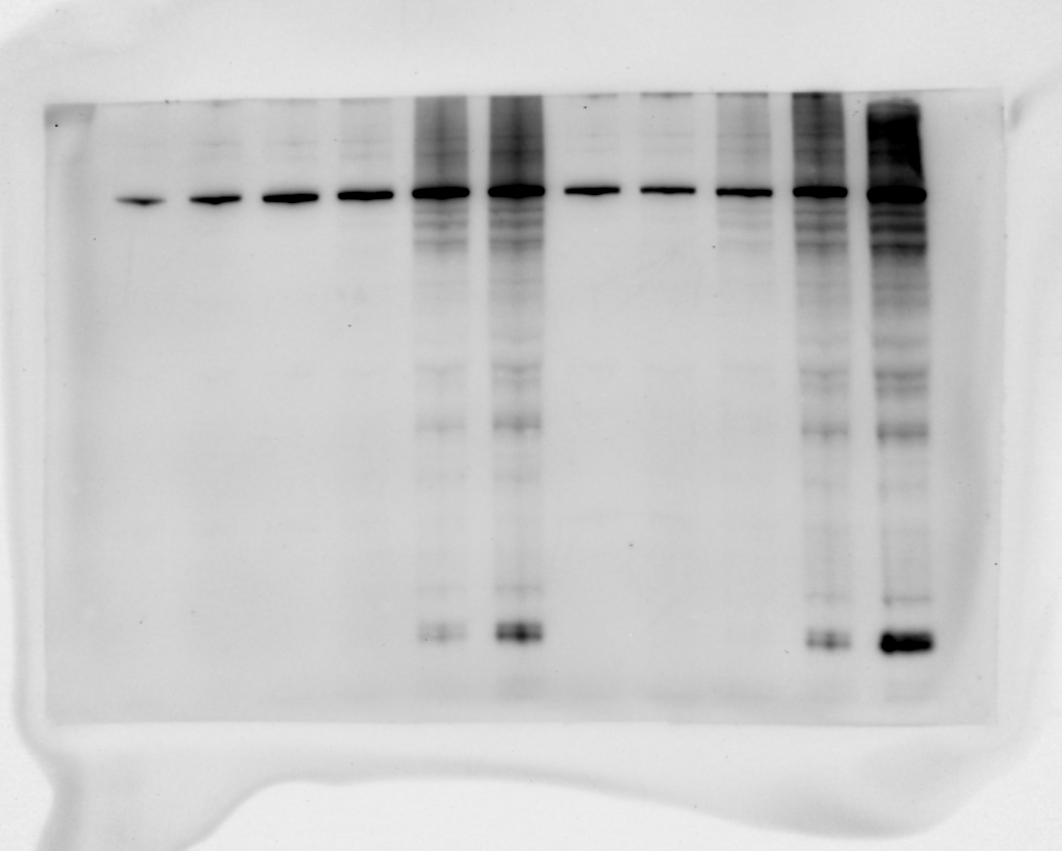

Supplement: Supplementary file 4 — Source data Fig. 6 [file 44319_2025_561_MOESM4_ESM.zip › 6E/CHEMIL_1.TIF]

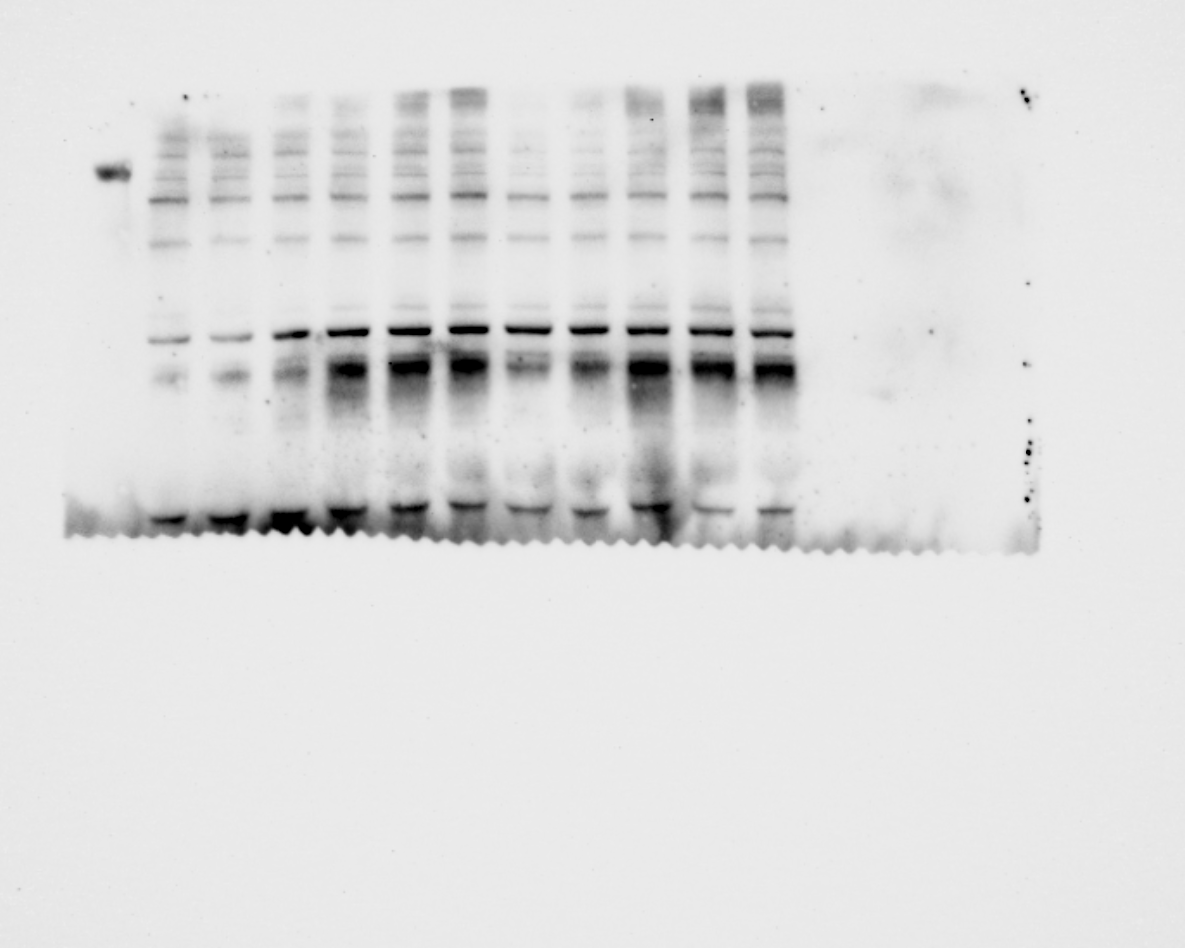

Supplement: Supplementary file 4 — Source data Fig. 6 [file 44319_2025_561_MOESM4_ESM.zip › 6E/Phospho_parkin_Ser65-Abcam.tif]

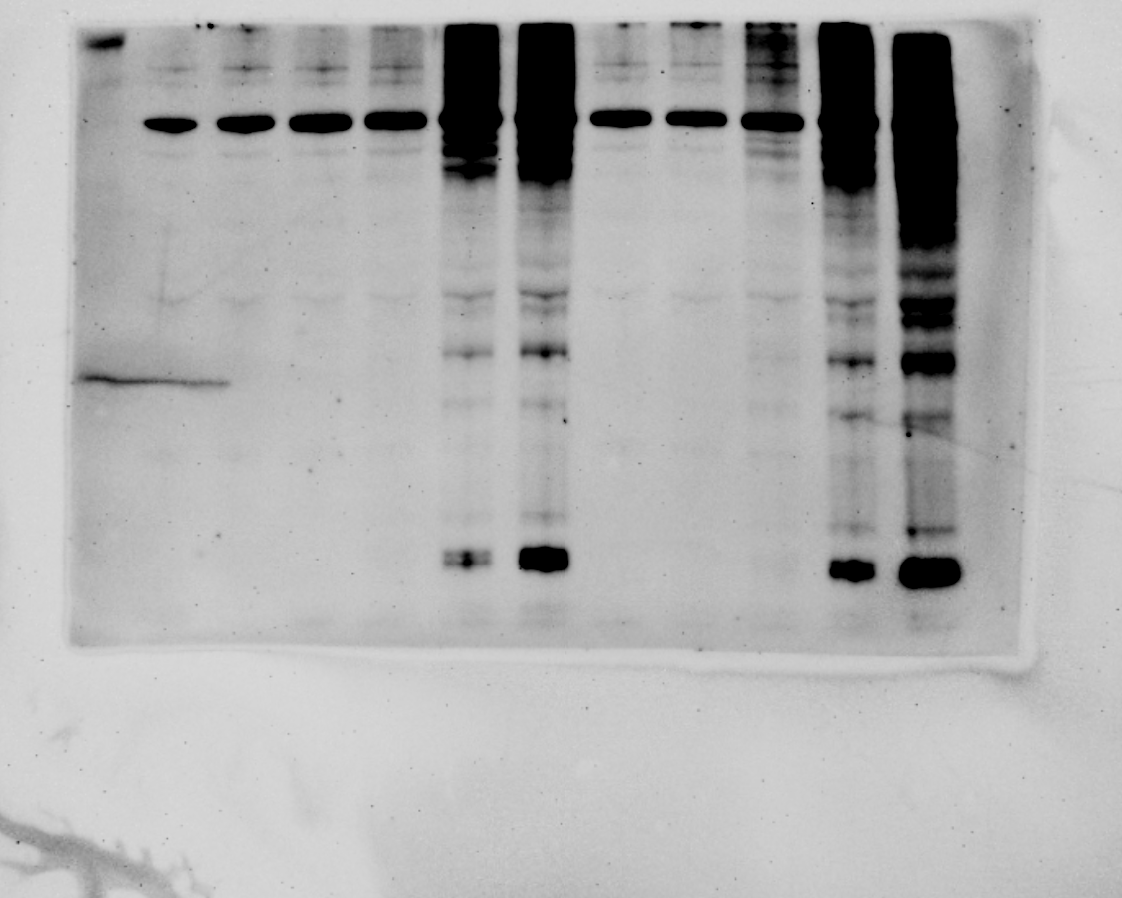

Supplement: Supplementary file 4 — Source data Fig. 6 [file 44319_2025_561_MOESM4_ESM.zip › 6E/Phospho_Ub_Ser65_Over_exposed.tif]

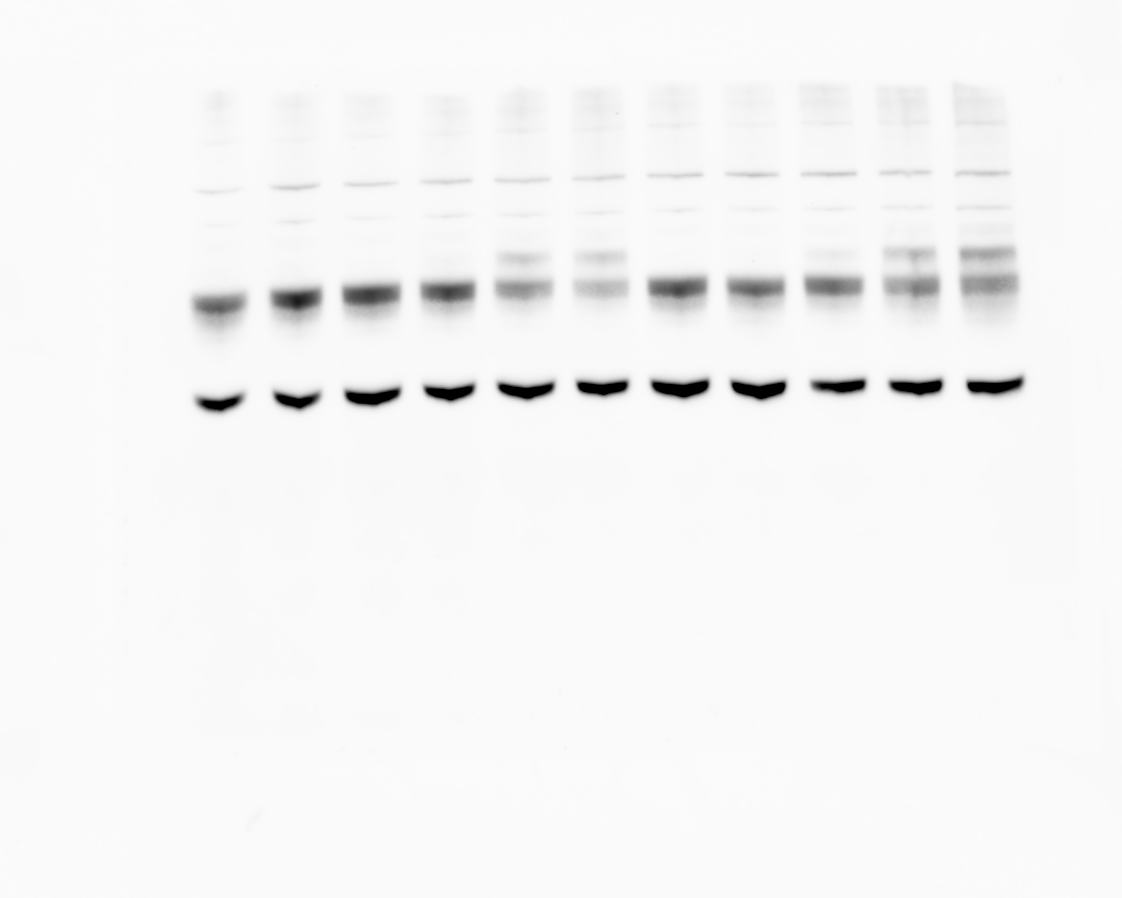

Supplement: Supplementary file 4 — Source data Fig. 6 [file 44319_2025_561_MOESM4_ESM.zip › 6E/Total_Parkin_Auto_exposed.tif]
