# Supplementary material for: Peri-mitochondrial actin filaments inhibit Parkin assembly by disrupting ER-mitochondria contacts
Source: EMBO Rep. 2025 Aug 29;26(20):4977–5008. doi: 10.1038/s44319-025-00561-y (PMC12550048; doi:10.1038/s44319-025-00561-y)
Supplement: Supplementary file 5 — Expanded View Figures [file 44319_2025_561_MOESM5_ESM.pdf]

Expanded View Figures

Figure EV1. ADA delays Parkin recruitment on depolarized mitochondria (CCCP treatment).

(**A**) RT-qPCR to validate knockdown of the respective genes in U2OS (upper; green bars) and HeLa cells (lower; red bars). Normalized expression data combined from 3 biological replicates. Error: Mean ±s.d. (**B**) Time lapse montages of WT HeLa cells transfected with GFP-Parkin (green) and Mito-BFP (red) pre-treated with 30 min 100 µM CK666 or DMSO before treatment with 20 µM CCCP treatment during live-cell imaging at time 0. Imaging conducted at the medial cell section. Scale: 10 µm. Time in minutes. (**C**) Quantification of colocalized Parkin signal with mitochondrial signal in live-cell imaging after 20 µM treatment of CCCP at time 0 for 30 min DMSO (black) or 100 µM CK666 (red) pre-treated HeLa cells. $N = 26$ cells for DMSO/CCCP and 31 for CK666/CCCP. 4 independent experiments. Error ± SEM. (**D**) Scatter plot of Parkin signal onset on mitochondria for DMSO or 100 µM CK666 treated HeLa cells. Parkin onset time = 52.64 min ± 11.35 (mean ± s.d.) for DMSO/CCCP and 25.23 ± 10.89 for CK666/CCCP. Same dataset as in Figure EV1C ($N = 26$ cells for DMSO/CCCP and 31 for CK666/CCCP. 4 independent experiments). Error ± s.d. $P = 0.0001$ (****). Student's unpaired *t* test used. (**E**) Time lapse montages of ctrl, Arp2 KD, and FMNL1/2 DKD HeLa cells transfected with GFP-Parkin (green) and Mito-BFP (red) treated with 20 µM CCCP treatment at time 0 during live-cell imaging. Imaging conducted at the medial cell section. Scale: 10 µm. Time in minutes. (**F**) Quantification of colocalized Parkin signal with mitochondrial signal in live-cell imaging after 20 µM treatment of CCCP at time 0 for ctrl (black), FMNL1/2 DKD (red) or Arp2 KD (gold) HeLa cells. Data of 38 cells for ctrl; 19 for FMNL1/2 DKD and 24 for Arp2 KD from 3 independent experiments. Error ± SEM. (**G**) Scatter plot of Parkin signal onset on mitochondria for ctrl, FMNL 1/2 DKD or Arp2 KD in HeLa cells. Parkin onset time = 46.32 min ± 12.16 (mean ± s.d.) for ctrl; 28.95 ± 6.12 for FMNL 1/2 DKD and 28.67 ± 6.01 for Arp2 KD cells. Same dataset as in Figure EV1F (Data of 38 cells for ctrl; 19 for FMNL1/2 DKD and 24 for Arp2 KD from 3 independent experiments). $P = 0.0001$ (****) for ctrl vs FMNL 1/2 DKD and ctrl vs Arp2 KD; $P = 0.9949$ (n.s.) for FMNL 1/2 DKD vs Arp2 KD. Tukey's multiple comparisons test used. (**H**) Representative fixed cell images of MEFs transfected with Parkin-GFP and mitoBFP treated with 20 µM CCCP in the presence or absence of CK666 (100 µM) for 60 min. Arrows show Parkin-GFP colocalized with the mitoBFP signal. Scale: 10 µm. (**I**) Quantification showing percentage of total mitochondrial area/cell positive for Parkin-GFP in MEFs treated with 20 µM CCCP in the presence or absence of CK666 (100 µM) for 60 min. MEFs treated with CCCP: 41 cells and MEFs treated with CCCP + CK666: 30 cells from 3 independent experiments. $P = 0.0001$ (****). Students *t* test used. Error: Mean ±s.d.

© The Author(s)

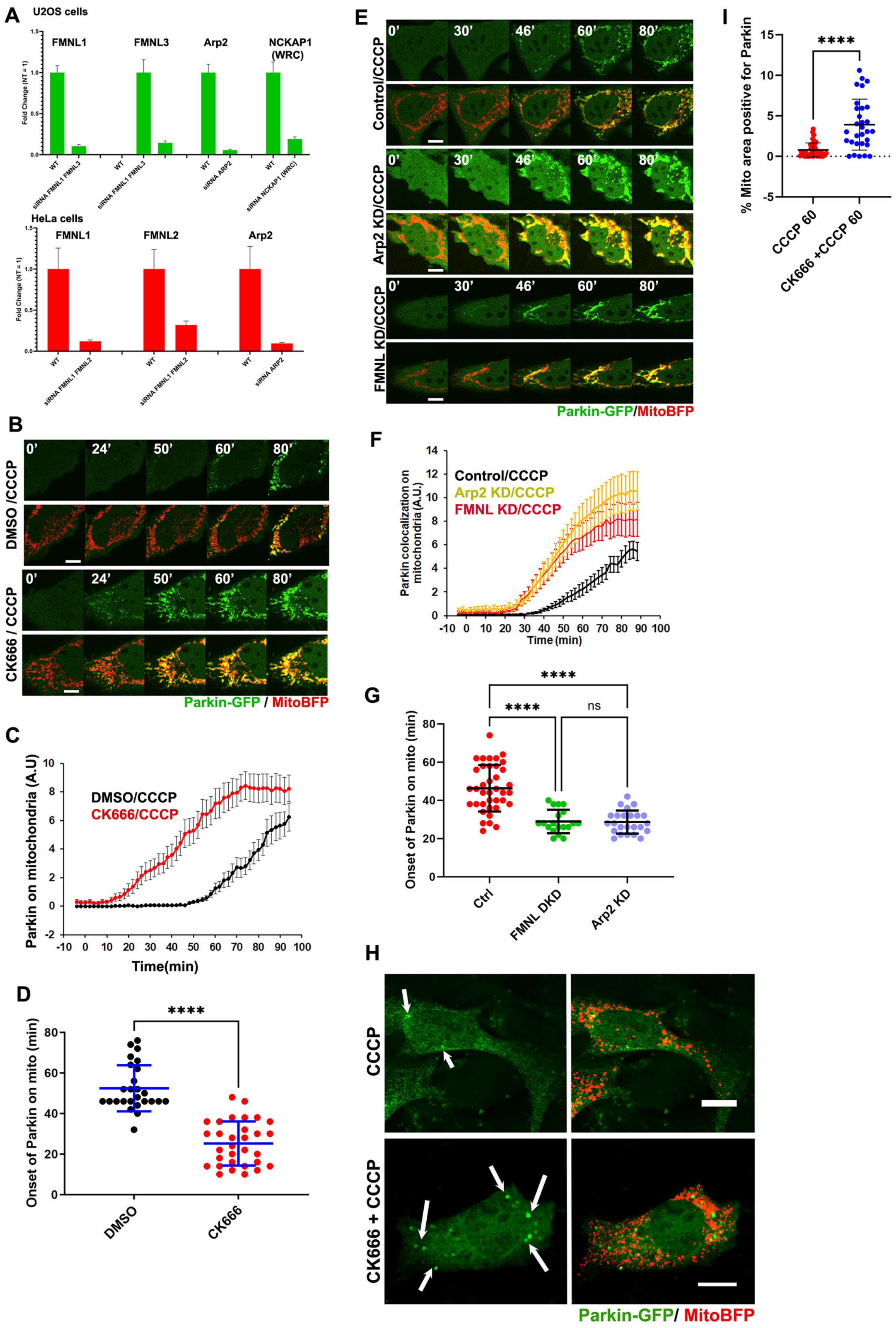
A
U2OS cells
FMNL1
FMNL3
Arp2
NCKAP1 (WRC)
Fold Change (NT = 1)
WT
siRNA FMNL1 FMNL3
siRNA ARP2
siRNA NCKAP1 (WRC)
HeLa cells
FMNL1
FMNL2
Arp2
siRNA FMNL1 FMNL2
B
DMSO /CCCP
CK666 / CCCP
0'
24'
50'
60'
80'
Parkin-GFP / MitoBFP
C
Parkin on mitochondria (A.U)
DMSO/CCCP
CK666/CCCP
Time(min)
D
Onset of Parkin on mito (min)
****
DMSO
CK666
E
Control/CCCP
Arp2 KD/CCCP
FMNL KD/CCCP
0'
30'
46'
60'
80'
Parkin-GFP/MitoBFP
F
Parkin colocalization on mitochondria (A.U.)
Control/CCCP
Arp2 KD/CCCP
FMNL KD/CCCP
Time (min)
G
Onset of Parkin on mito (min)
****
****
ns
Ctrl
FMNL DKD
Arp2 KD
H
CCCP
CK666 + CCCP
Parkin-GFP/ MitoBFP
I
% Mito area positive for Parkin
****
CCCP 60
CK666 +CCCP 60

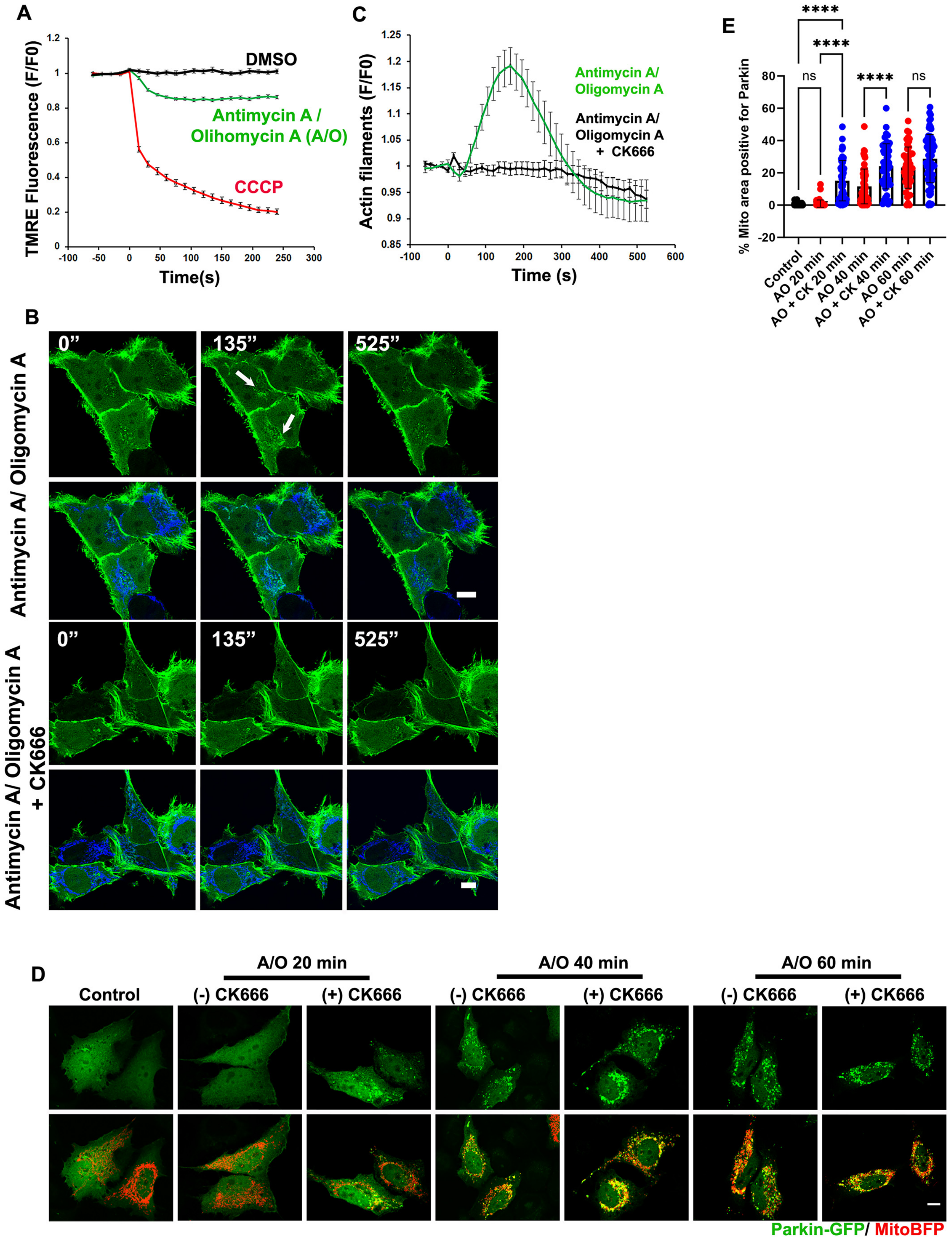
A
TMRE Fluorescence (F/F0)
Time(s)
DMSO
Antimycin A /
Olihomycin A (A/O)
CCCP
C
Actin filaments (F/F0)
Time (s)
Antimycin A/
Oligomycin A
Antimycin A/
Oligomycin A
+ CK666
E
% Mito area positive for Parkin
Control
AO 20 min
AO + CK 20 min
AO 40 min
AO + CK 40 min
AO 60 min
AO + CK 60 min
ns
****
B
Antimycin A/ Oligomycin A
Antimycin A/ Oligomycin A
+ CK666
0”
135”
525”
D
Control
A/O 20 min
A/O 40 min
A/O 60 min
(-) CK666
(+) CK666
Parkin-GFP/ MitoBFP

 © The Author(s)

Figure EV2. Antimycin A/Oligomycin A induced ADA delays Parkin recruitment in HeLa cells.

(**A**) Graph showing kinetics of TMRE fluorescence in HeLa cells treated with either DMSO (black) or 25 μM Antimycin A/ 15 μM Oligomycin A (A/O) or 20 μM CCCP. Treatment added at time 0. Data from 3 independent experiments totaling 25 cells (DMSO), 39 cells (CCCP) and 42 cells (A/O). Error: ±SEM. (**B**) Time-lapse montage of HeLa cells transfected with GFP-Ftractin and mitoBFP treated with 25 μM Antimycin A/ 15 μM Oligomycin A (A/O) in the presence or absence of CK666 (100 μM) at time 0 min. Arrows show the induction of mitochondrial-associated actin. Scale bar: 10 μm. (**C**) Graph showing actin filaments fluorescence quantified from the data set in Fig EV2B. Treatment added at time 0. Data combine 3 independent experiments having 21 cells (A/O) and 29 cells (A/O + CK666). Error: Mean ±SEM. (**D**) Representative fixed cell images showing HeLa cells transfected with Parkin-GFP and mitoBFP and treated with 25 μM Antimycin A/ 15 μM Oligomycin A (A/O) in the presence or absence of CK666 (100 μM) for various time points as indicated. (**E**) Scatter plot showing percentage of total mitochondrial area/cell positive for Parkin-GFP in HeLa cells treated with 25 μM Antimycin A/ 15 μM Oligomycin A (A/O) in the presence or absence of CK666 (100 μM) for various time points. Data from 3 independent experiments having Control: 62 cells; A/O 20 min: 53 cells; A/O + CK666 20 min: 57 cells; A/O 40 min: 64 cells; A/O + CK666 40 min: 50 cells; A/O 60 min: 47 cells; A/O + CK666 60 min: 69 cells. $P = 0.0001$ (****). Students *t* test used. Error ± s.d.

© The Author(s)

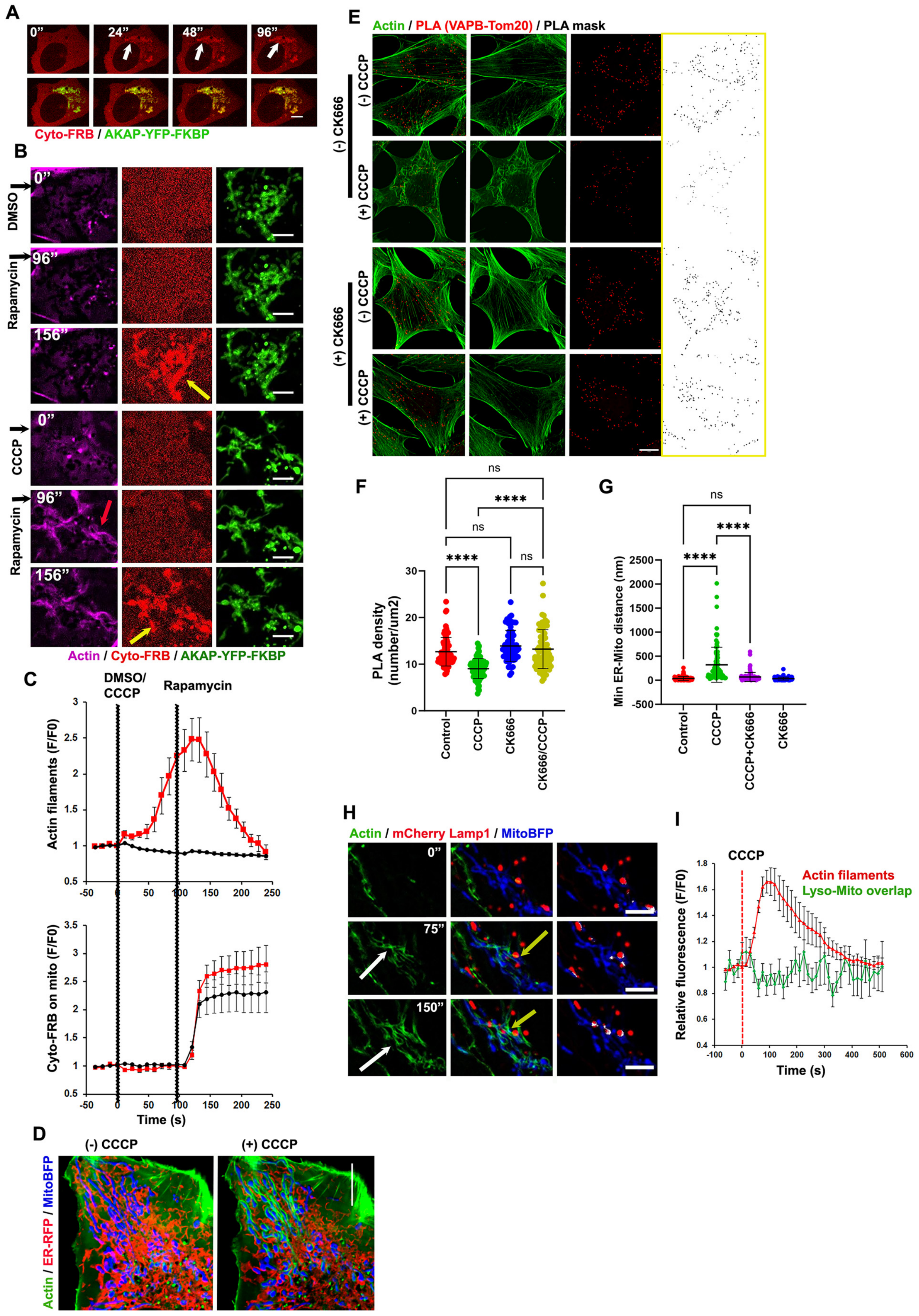
A
0”
24”
48”
96”
Cyto-FRB / AKAP-YFP-FKBP
B
DMSO
Rapamycin
CCCP
Rapamycin
0”
96”
156”
Actin / Cyto-FRB / AKAP-YFP-FKBP
C
DMSO/ CCCP
Rapamycin
Actin filaments (F/F0)
Cyto-FRB on mito (F/F0)
Time (s)
D
(-) CCCP
(+) CCCP
Actin / ER-RFP / MitoBFP
E
Actin / PLA (VAPB-Tom20) / PLA mask
(-) CK666
(+) CK666
(-) CCCP
(+) CCCP
F
PLA density (number/um2)
Control
CCCP
CK666
CK666/CCCP
ns
****
G
Min ER-Mito distance (nm)
Control
CCCP
CCCP+CK666
CK666
H
Actin / mCherry Lamp1 / MitoBFP
0”
75”
150”
I
CCCP
Actin filaments
Lyso-Mito overlap
Relative fluorescence (F/F0)
Time (s)

 © The Author(s)

Figure EV3. ADA disrupts ER-mitochondrial contacts but not mitochondria-lysosome contacts.

(**A**) Time lapse montage of U2OS cells transfected with cyto-(FRB)5 and AKAP1-YFP-FKBP and treated with 10 μM Rapamycin at time 0 as indicated. Scale bar: 5 μm. (**B**) Time lapse montage of U2OS cells transfected with cyto-(FRB)5, AKAP1-YFP-FKBP CCCP and GFP-Ftractin and treated with either DMSO or CCCP for 100 s followed by rapamycin (final 10 μM). White arrows indicate Cyto-FRB positive mitochondria. (**C**) Quantification of actin filaments (upper) and mitochondrially associated CFP-(FRB)5 (lower) after the following treatments: DMSO or CCCP at time 0 followed by rapamycin (final 10 μM) treatment after 100 s of initial treatment. Data from 9 cells for each condition from 3 independent runs. Error: ±SEM. (**D**) Micrographs from U2OS cells transfected with ER-RFP (ER; red), mito-BFP (mitochondria; blue) and GFP-F tractin (actin filaments; green) before and after 100 s of CCCP treatment. Scale bar: 5 μm. (**E**) Representative fixed-cell images from Proximity Ligation Assay (PLA) conducted between VAPB (ER) and Tom20 (mitochondria) to assess ERMC (red and binarized) in HeLa cells treated with 20 μM CCCP for 5 min in the presence or absence of CK666 (100 μM). Cells were stained with Phalloidin-488 to label actin filaments prior to PLA staining. Scale: 10 μm. (**F**) Scatter plot representing density of PLA dots (no. per 100 μm^2) in control and CCCP-treated HeLa cells in the presence or absence of CK666. Same data set as Fig EV3E. Data with 80 cells (control); 82 cells (CCCP); 65 cells (CK666) and 77 cells (CK666 + CCCP) from 4 independent coverslips. *P* = 0.0001 (****). Two-way ANOVA used. Error: Mean ±s.d. (**G**) Scatter plot showing the minimum distance between ER and mitochondria as estimated from TEM images represented in data set in Fig. 2I–J (Control *n* = 88 mitochondria; 20 μM CCCP/ 5 min: *n* = 94 mitochondria; 20 μM CCCP + CK666 (100 μM/ 5 min); *n* = 90 mitochondria and 100 μM CK666 treated; *n* = 106 mitochondria) in MEF cells. Data from three independent fixation for each condition. Error: ±s.d.; *P* = 0.0001 (****). Two-way ANOVA used. (**H**) Time-lapse montages of CCCP-induced actin polymerization and lysosome-mitochondria overlap in U2OS cells transfected with mCherry-Lamp1 (lysosomes; red), mito-BFP (mitochondria; green) and GFP-F tractin (actin filaments; magenta). White arrow indicates mitochondria-lysosome contact. Scale bar: 5 μm. (**I**) Quantification of CCCP-induced actin polymerization and lysosome-mitochondria overlap in U2OS cells transfected with mCherry-Lamp1 (lysosome; red), mito-BFP (mitochondria; green) and GFP-F tractin (actin filaments; magenta) 20 cells for each condition obtained from 2 independent experiments. Error: ±SEM.

© The Author(s)

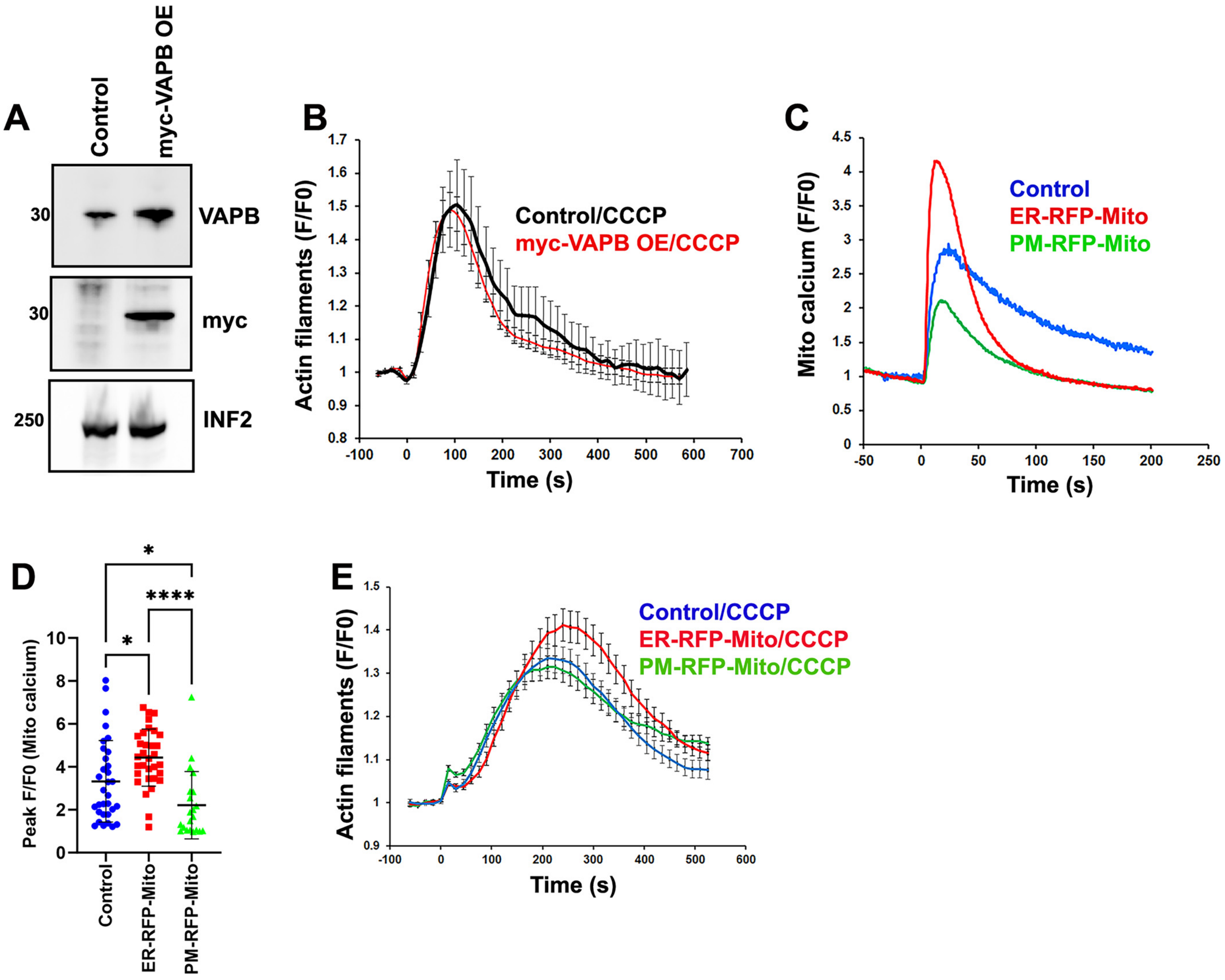

Figure EV4. ERMC regulates CCCP-induced Parkin recruitment.

(**A**) Western blot for VAP-B and myc-tag showing VAP-B expression in control and myc-VAP-B overexpressed U2OS cells. INF2 is used as loading control. Molecular weight in kDa. (**B**) Quantification of CCCP-induced actin polymerization in control and myc-VAP-B-overexpressing U2OS cells. Data with 65 cells (control) and 53 cells (overexpressing myc-VAP-B) from 3 independent runs. Error ± SEM. (**C**) Averaged traces showing mitochondrial calcium fold change (MitoGCamp6f) following histamine stimulation (at time 0) in control (ER-RFP expressing), ER-RFP-mito (ER-Mito linker) and PM-RFP-Mito (PM-Mito-linker) transfected HeLa cells. Data from 2 independent experiments comprising of 4 individual traces for each condition. Error: ±SEM. (**D**) Dot plot of peak fold change in mitochondrial calcium following histamine stimulation from individual traces as in Fig S4C. Data for 32 cells (Control- ER-RFP); 32 cells (ER-RFP-Mito); 28 cells (PM-RFP-Mito) for 3 independent runs. Error: ±s.d. $P = 0.0213$ (*) for Control Vs ER-RFP-Mito, $P = 0.0439$ (*) for Control Vs PM-RFP-Mito and $P = 0.0001$ (****) for ER-RFP-Mito Vs PM-RFP-Mito, One-way ANOVA used. (**E**) Graph showing actin filaments intensity (GFP-Ftractin) from HeLa cells transiently expressing either ER-RFP-Mito (magenta) or PM-RFP-Mito or ER-RFP, treated with 20 μM CCCP. Data from 28 cells (ER-RFP); 27 cells (ER-RFP-Mito) and 23 cells (PM-RFP-Mito) from 4 independent experiments. Error: ±SEM.

 © The Author(s)

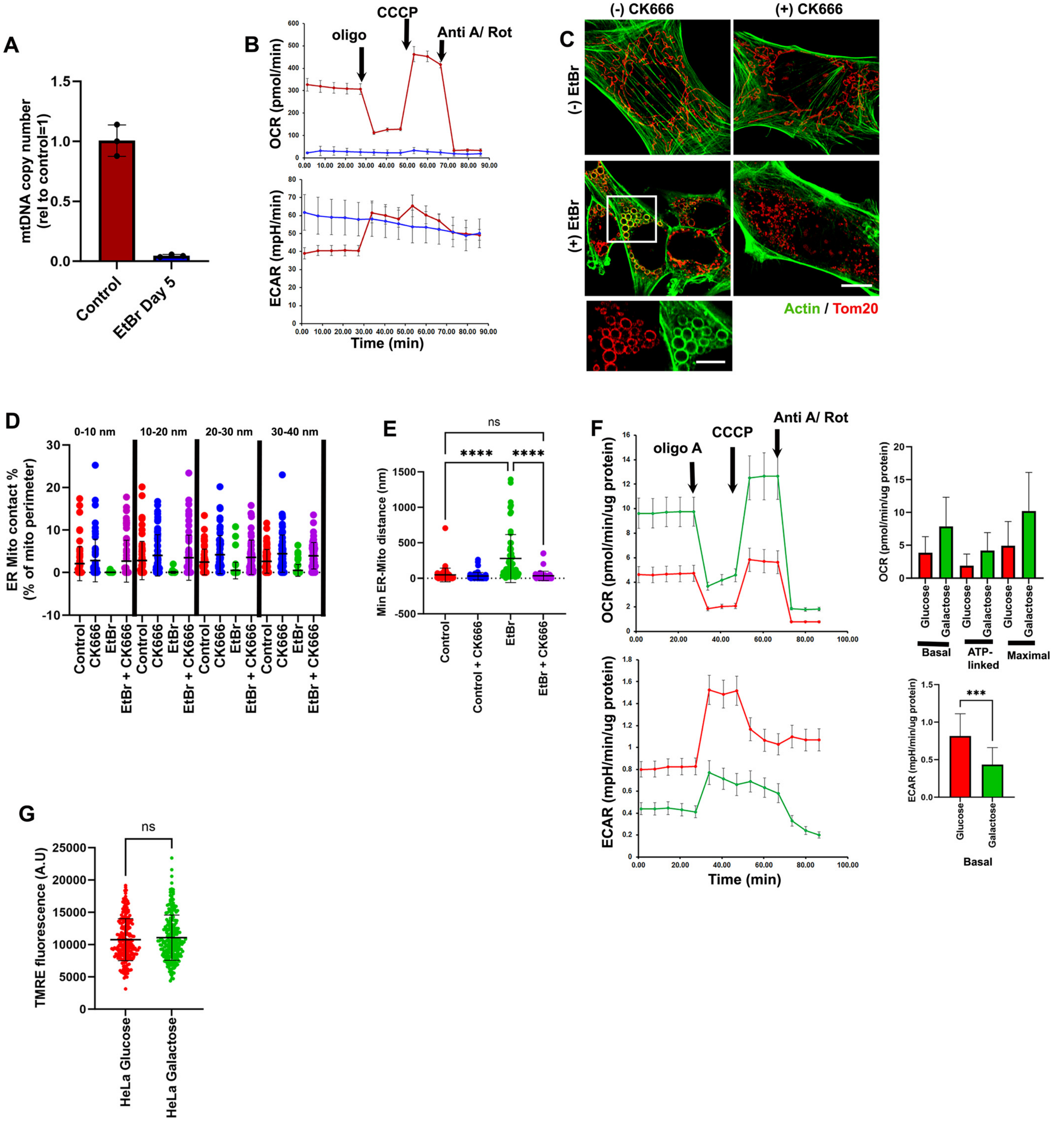
A
mtDNA copy number (rel to control=1)
Control
EtBr Day 5
B
oligo
CCCP
Anti A/ Rot
OCR (pmol/min)
ECAR (mpH/min)
Time (min)
C
(-) CK666
(+) CK666
(-) EtBr
(+) EtBr
Actin / Tom20
D
0-10 nm
10-20 nm
20-30 nm
30-40 nm
ER Mito contact % (% of mito perimeter)
Control
CK666
EtBr
EtBr + CK666
E
ns
****
****
Min ER-Mito distance (nm)
Control
Control + CK666
EtBr
EtBr + CK666
F
oligo A
CCCP
Anti A/ Rot
OCR (pmol/min/ug protein)
ECAR (mpH/min/ug protein)
Time (min)
Glucose
Galactose
Basal
ATP-linked
Maximal
***
G
ns
TMRE fluorescence (A.U)
HeLa Glucose
HeLa Galactose

Figure EV5. EtBr-induced mtDNA depletion & formation of ADA-like filaments and OxPhos dependency causes prolonged ADA.

(**A**) RT-qPCR to validate depletion of mitochondrial DNA (mtDNA) in MEFs treated with and without EtBr (0.2 μg/ml) for 5 days. Normalized expression data derived from 3 biological replicates. $P = 0.0001$ (****). Student's *T* test used. Error: Mean ±s.d. (**B**) Representative traces of OCR and ECAR readout from MEFs treated with and without EtBr (0.2 μg/ml) for 5 days. Error: ±s.d. $N=$ at least 3 independent wells. (**C**) MEFs treated with or without EtBr (0.2 μg/ml) for 5 days, treated with CK666 for 4 h (100 μM), fixed, and stained for actin filaments (green) and mitochondria (red). Scale bars: 10 μm (main panel); 5 μm (inset). (**D**) Scatter plot showing the percentage of mitochondrial perimeter within 0–40 nm distance distributed within bins of 10 nm; Same data set as in Fig. 7D. Control (Red; $n = 62$ mitochondria); Control with CK666 (green; $n = 63$ mitochondria); EtBr (purple; $n = 63$ mitochondria) EtBr with CK666 (blue; $n = 65$ mitochondria). Data from three independent fixation for each condition; Error: Mean ±s.d. (**E**) Scatter plot showing the minimum distance between ER and mitochondria as estimated from TEM images represented in dataset from Fig. 7C, D (Controls without CK666; $n = 62$ mitochondria; with CK666; $n = 63$ mitochondria; +EtBr without CK666: $n = 63$ mitochondria and +EtBr with CK666: $n = 65$ mitochondria). Three independent experiments for each condition $P = 0.0001$ (****). One-way ANOVA used. Error: Mean ±s.d. (**F**) OCR and ECAR traces of HeLa cells cultured in either 10 mM glucose or galactose for 10 days, sequentially treated with oligomycin (1.5 μM), FCCP (1 μM) and Antimycin/Rotenone (2.5 μM/ 1 μM) at designated times. Bar graph showing basal, ATP-linked, and maximal respiration calculated from traces shown. Error ± s.d. $P = 0.0041$ (**), 0.0112 (*) and 0.0056 (***), student's *t* test used. Bar graph showing ECAR rates (glycolysis) in unstimulated HeLa cells cultured in glucose or galactose for 10 days from traces shown. $P = 0.0003$ (***), student's *t* test used. Error: Mean ±s.d. (**G**) Dot plot showing TMRE fluorescence (measuring mitochondrial membrane potential) in HeLa cells cultured in either 10 mM glucose or galactose for 10 days. Data from 213 cells (HeLa glucose) and 228 cells (HeLa galactose) from 4 independent experiments. Student's *t* test used. Error: Mean ±s.d.

 © The Author(s)
